# Reply: Cancer risk before and following organ transplantation

**DOI:** 10.1038/sj.bjc.6602015

**Published:** 2004-07-13

**Authors:** J Adami

**Affiliations:** 1 Department of Medical Epidemiology and Biostatistics, Karolinska Institutet, M9:01, Stockholm SE-17176, Sweden

**Sir**,

We thank doctors Maisonneuve and Lowenfels for their interest in our work and for their insightful comments. We fully agree that in a record linkage study with limited covariate information, it is impossible to clearly distinguish the effects on the underlying disease from the effects of organ transplantation and subsequent immunosuppression. As a corollary, we are presently conducting a series of nested case–control studies within the cohort of organ-transplanted patients. This will allow us to more directly quantify the effect of both indication for organ transplantation and subsequent immunosuppression and other pharmacologic treatment.

There are rather few population-based epidemiological studies that have explored the relationship between end-stage renal disease and subsequent development of cancer. There seems to be a convincing positive association with cancer of the kidney and bladder. However, in our view, the risk for other malignancies associated with end-stage renal disease and its different causes remains to be adequately quantified, since previous studies show somewhat conflicting results and is hampered by inadequate power. Another evidence of the impact of organ transplantation and immune suppressive drugs is the even more pronounced effects following transplantation of organs other than the kidney. Also, the relative risk estimates are in general much higher following organ transplantation compared to end-stage renal disease, suggesting other mechanisms as well.

Lastly, Maisonneuve and Lowenfels suggest that the differential misclassification of cervical cancer in the transplantation cohort compared with the background population accounts for the absence of excess of cervical cancer. We consider this explanation unlikely, and the SIR for uterus unspecified supports this view.

